# Association of CSF GAP-43 and *APOE* ε4 with Cognition in Mild Cognitive Impairment and Alzheimer’s Disease

**DOI:** 10.3390/cells12010013

**Published:** 2022-12-21

**Authors:** Yueli Zhu, Xiaoming Guo, Feng Zhu, Qin Zhang, Yunmei Yang, for the Alzheimer’s Disease Neuroimaging Initiative

**Affiliations:** 1 Department of Geriatrics, The First Affiliated Hospital, School of Medicine, Zhejiang University, Hangzhou 310003, China; 2 Department of Neurosurgery, Tongde Hospital of Zhejiang Province, Hangzhou 310012, China; 3 Key Laboratory of Diagnosis and Treatment of Aging and Physic-Chemical Injury Diseases of Zhejiang Province, The First Affiliated Hospital, School of Medicine, Zhejiang University, Hangzhou 310003, China

**Keywords:** Alzheimer’s disease, growth-associated protein 43, Apolipoprotein E ε4, synaptic loss, biomarker

## Abstract

The growth-associated protein 43 (GAP-43) is a presynaptic phosphoprotein in cerebrospinal fluid (CSF). The ε4 allele of apolipoprotein E (*APOE*) is an important genetic risk factor for Alzheimer’s disease (AD). We aimed to evaluate the association of CSF GAP-43 with cognition and whether this correlation was related to the *APOE* ε4 status. We recruited participants from the Alzheimer’s Disease Neuroimaging Initiative (ADNI) database, and they were divided into cognitively normal (CN) ε4 negative (CN ε4−), CN ε4 positive (CN ε4+), mild cognitive impairment (MCI) ε4 negative (MCI ε4−), MCI ε4 positive (MCI ε4+), AD ε4 negative (AD ε4−), and AD ε4 positive (AD ε4+) groups. Spearman’s correlation was utilized to evaluate the relationship between CSF GAP-43 and core AD biomarkers at the baseline. We performed receiver-operating characteristic (ROC) curve analyses to investigate the diagnostic accuracy of CSF GAP-43. The correlations between CSF GAP-43 and the Mini-Mental State Examination (MMSE) scores and brain atrophy at baseline were assessed by using multiple linear regression, while the association between CSF GAP-43 and MMSE scores at the follow-up was tested by performing the generalized estimating equation (GEE). The role of CSF GAP-43 in the conversion from MCI to AD was evaluated using the Cox proportional hazard model. We found that the CSF GAP-43 level was significantly increased in MCI ε4+, AD ε4− and AD ε4+ groups compared with CN ε4− or MCI ε4− group. The negative associations between the CSF GAP-43 and MMSE scores at the baseline and follow-up were found in MCI ε4− and MCI ε4+ groups. In addition, baseline CSF GAP-43 was able to predict the clinical progression from MCI to AD. CSF GAP-43 may be a promising biomarker to screen cognition for AD. The effects of CSF GAP-43 on cognition were suspected to be relevant to *APOE* ε4 status.

## 1. Background

Alzheimer’s disease (AD) is the most common type of dementia presenting with progressive cognitive decline and is characterized by abnormal accumulation of extracellular amyloid-β (Aβ) plaques, intracellular neurofibrillary tangles of tau protein, and neurodegeneration [1,2]. As we know, pathological changes in the brains of AD patients occur prior to the onset of clinical symptoms. Therefore, it is critical to identify patients early and provide timely intervention. According to the newly updated AT(N) research framework, three groups of biomarkers are recognized, including aggregated Aβ or associated pathophysiologic processes (labeled “A”); aggregated tau or associated pathophysiologic processes (labeled “T”); and neurodegeneration or neuronal injury (labeled “N”) [3]. Furthermore, this AT(N) scheme is also designed to incorporate new biomarkers to better reflect the mechanisms of AD [3].

Synapse dysfunction and loss are prominent neuropathological features of AD, which occur early even at the stage of mild cognitive impairment (MCI) [4]. The loss of synapses is considered to be associated with the degree of cognitive impairment in AD patients [5]. The growth-associated protein 43 (GAP-43) is a presynaptic phosphoprotein and has been found to be neuron-specific and important for modulating synaptic functions as required for learning and memory [6,7,8]. There is evidence showing that the level of cerebrospinal fluid (CSF) GAP-43 increases even in the early stage of AD [9,10,11]. The increase in GAP-43 levels may be attributed to the leakage of GAP-43 into CSF as a consequence of the adaptive response to synaptic degradation [10]. The ε4 allele of the *APOE* gene remains to be the strongest genetic risk factor for AD since it was found in 1993 [12,13,14]. It has been reported that *APOE* ε4 targeted replacement mice have increased the levels of the vesicular glutamate transporter VGLUT1, indicating that the *APOE* genotype can affect the presynaptic terminal composition and consequently contribute to neurodegeneration [15].

Hence, we wonder about the potential role of CSF GAP-43 for AD and whether *APOE* ε4, which has been found to exert effects on presynaptic function, could influence CSF GAP-43 levels. In this present study, we mainly investigated the effects of CSF GAP-43 on cognitive impairment and whether the roles of GAP-43 were related to *APOE* ε4 status among cognitively normal (CN) controls, MCI and AD participants from the Alzheimer’s Disease Neuroimaging Initiative (ADNI) database.

## 2. Methods

### 2.1. ADNI Database Description

All data were obtained from the ADNI database (adni.loni.usc.edu), a public–private partnership launched in 2003 under the leadership of Principal Investigator Michael W. Weiner, MD. The primary goal of ADNI has been to test the roles of magnetic resonance imaging (MRI), positron emission tomography (PET), CSF and blood biological markers, and neuropsychological assessments in detecting the progression of MCI and early AD. For more information, see www.adni-info.org, accessed on 25 October 2021. 

### 2.2. Participants and Classification Criteria

From the ADNI database, we recruited participants with available CSF core biomarkers for AD and GAP-43 levels, Mini-Mental State Examination (MMSE) scores, Clinical Dementia Rating (CDR) scales, and MRI imaging. These selected participants were classified as CN (*n* = 238), MCI (*n* = 388), and AD (*n* = 118) groups on the basis of diagnoses provided by the ADNI database. Participants with at least one ε4 allele were defined as *APOE* ε4 carriers. They were further divided into six groups based on *APOE* ε4 status: CN ε4 negative (CN ε4−, *n* = 169); CN ε4 positive (CN ε4+, *n* = 69); MCI ε4 negative (MCI ε4−, *n* = 204); MCI ε4 positive (MCI ε4+, *n* = 184); AD ε4 negative (AD ε4−, *n* = 40); and AD ε4 positive (AD ε4+, *n* = 78) groups.

CN participants were defined as those who had an MMSE score between 24 and 30 and a CDR score of 0 [16]. Participants with MCI had an MMSE score of 24 to 30, a CDR score of 0.5, subjective complaints of memory, and remaining activities of daily living; therefore, the diagnosis of dementia cannot be made [16]. AD participants fulfilled the criteria of NINCDS/ADRDA for probable AD, who had an MMSE score between 20 and 26 and a CDR score of 0.5 or 1.0 [16,17].

### 2.3. CSF Measurements

The levels of CSF Aβ42, total tau (T-tau), and phosphorylated tau at threonine 181(P-tau) were tested using the multiplex xMAP Luminex platform (Luminex Corp, Austin, TX, USA) with Innogenetics (INNO-BIA AlzBio3; Ghent, Belgium) immunoassay kit-based reagents [18]. 

CSF GAP-43 was analyzed by enzyme-linked immunoassay (ELISA) technology, using an in-house ELISA method described previously in detail [19]. The mouse monoclonal GAP-43 antibody NM4 (Fujirebio, Ghent, Belgium) and a polyclonal GAP-43 antibody (ABB-135, Nordic Biosite, Täby, Sweden) were combined in the ELISA procedure. Board-certified laboratory technicians performed these analyses. Residual CSF samples were used for quality control (QC1 and QC2). During sample runs in the clinical evaluation study, the repeatability coefficient of variation (CV)% of QC1 and QC2, was 5.5% versus 11% and the inter-assay CV% was 6.9% versus 15.6%. All values were given as pg/mL.

### 2.4. Neuroimaging Methods and Cognitive Assessments

Hippocampal and ventricular volumes adjusted by the intracranial volumes were selected for further analyses. The neuroimaging methods from the ADNI database have been described in detail previously [20]. We used the MMSE scores to assess the global cognitive function. Six time points of MMSE scores were analyzed, including baseline, 6, 12, 24, 36, and 48 months.

### 2.5. Statistical Methods

Since the baseline demographic data and biomarker levels were not normally distributed, the differences among three or more independent groups were compared using the Chi-square test for categorical variables and the Kruskal–Wallis test for continuous variables which were summarized with median and interquartile range (IQR). The Mann–Whitney U test was used to examine the differences in CSF GAP-43 levels between MCI ε3/ε4 and MCI ε4/ε4 groups, as well as between AD ε3/ε4 and AD ε4/ε4 groups. The Spearman’s correlation test was applied to assess the correlations between CSF GAP-43 levels and CSF core AD biomarkers, including CSF Aβ42, T-tau, and P-tau.

For comparing the diagnostic accuracy of each biomarker, the area under the curves (AUCs) with 95% confidence intervals (CIs) were calculated by receiver operating characteristic (ROC) curve analyses. Age, sex, and education were adjusted in all ROC curves. The differences between the AUCs of two biomarkers or combinations were tested by using MedCalc Statistical Software version 20.019.

The correlations between CSF GAP-43 levels and MMSE scores, hippocampal volumes, and ventricular volumes at baseline were evaluated with multiple linear regression (adjustment for age and sex; for education for MMSE; and for intracranial volumes for hippocampal and ventricular volumes). CSF GAP-43, MMSE scores, hippocampal volumes, ventricular volumes, and intracranial volumes were z-scale transformed to ensure normality. The influence of CSF GAP-43 levels on longitudinal cognition was tested using the generalized estimating equation (GEE), which accounted for the possible correlation of variables measured in the same participant over time and allowed for missing values [21]. In this study, MMSE scores at different follow-up time points were modeled as dependent variables while baseline CSF GAP-43 level was modeled as independent variables in GEE analyses, after adjustment for age, sex, and education.

The influence of GAP-43 on the incidence of MCI conversion to AD was assessed by using Cox proportional hazard model and calculating hazard ratio (HR) with 95% CIs, after adjustment for age and sex and education. MCI participants were divided into two groups according to the median value of GAP-43 levels. The statistical significance level of all analyses was defined as *p* < 0.05. All statistical analyses were performed using IBM SPSS Statistics version 26 and GraphPad Prism version 8.0.1. 

## 3. Results

### 3.1. CSF GAP-43 Levels in Different Diagnostic Groups

**Figure 1:**
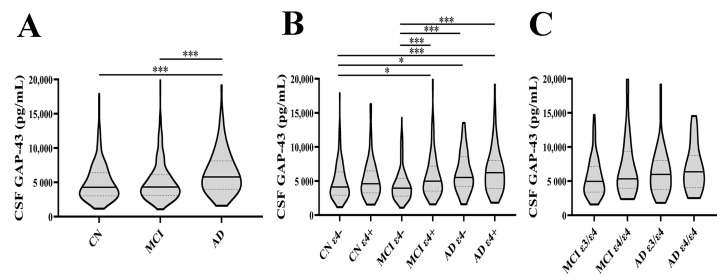
Differences in CSF GAP-43 levels among CN, MCI, and AD groups were tested using Kruskal–Wallis test (**A**). Differences in CSF GAP-43 levels among CN ε4−, CN ε4+, MCI ε4−, MCI ε4+, AD ε4− and AD ε4+ groups were evaluated using the Kruskal–Wallis test (**B**). Differences in CSF GAP-43 levels between MCI ε3/ε4 and MCI ε4/ε4 groups, as well as between AD ε3/ε4 and AD ε4/ε4 groups were assessed using the Mann–Whitney U test (**C**). *: *p* < 0.05; ***: *p* < 0.001. CSF: cerebrospinal fluid; GAP-43: growth-associated protein 43; CN: cognitively normal; MCI: mild cognitive impairment; AD: Alzheimer’s disease.

We included a total of 744 participants from the ADNI database (adni.loni.usc.edu). All of these individuals were divided according to the diagnoses and *APOE* ε4 status. CSF GAP-43 levels were significantly higher in the AD group than in CN and MCI groups (both *p* < 0.001) (Figure 1A). Compared with CN ε4− group, higher CSF GAP-43 levels were observed in MCI ε4+, AD ε4−, and AD ε4+ groups (*p* = 0.045, *p* = 0.027, *p* < 0.001, respectively) (Figure 1B). Compared with the MCI ε4− group, MCI ε4+, AD ε4−, and AD ε4+ groups also had significantly higher GAP-43 levels (all *p* < 0.001) (Figure 1B). We further investigated the differences between carriers of one or two ε4 alleles and the CN group was not analyzed since the carriers of two ε4 alleles in this group were rare. It seemed that the level of GAP-43 in the MCI ε4/ε4 group was higher than MCI ε3/ε4 group; however, no significant difference was found (*p* = 0.094) (Figure 1C). There was a similar phenomenon between AD ε3/ε4 and AD ε4/ε4+groups (*p* = 0.506) (Figure 1C).

### 3.2. Characteristics of Included Participants

The demographics and biomarker features of the study participants were provided in Table 1. We found a statistically significant difference in age among the six groups (*p* < 0.001). As for sex, there were more female participants in the CN ε4+ group than the AD ε4−group (*p* = 0.013). No statistical difference was observed among the six groups for education (*p* = 0.131). Six groups differed significantly in terms of MMSE scores and CSF biomarkers. The CN ε4− and CN ε4+ groups had higher MMSE scores than MCI ε4− and MCI ε4+ groups, which had higher MMSE scores than AD ε4− and AD ε4+ groups. The levels of CSF Aβ42 were much lower in *APOE* ε4 positive participants between CN ε4− and CN ε4+ groups (*p* < 0.001), between MCI ε4− and MCI ε4+ groups (*p* < 0.001), and between AD ε4− and AD ε4+ groups (*p* = 0.031). T-tau level was significantly higher in the MCI ε4+ group compared with the MCI ε4− group (*p* < 0.001). However, T-tau levels showed no significant differences between CN ε4− and CN ε4+ groups, or between AD ε4− and AD ε4+ groups. Between CN ε4- and CN ε4+ groups (*p* = 0.003) and between MCI ε4− and MCI ε4+ groups (*p* < 0.001), the levels of P-tau were much higher in the *APOE* ε4 carriers. However, a similar phenomenon was not observed between AD ε4− and AD ε4+ groups.

### 3.3. Correlations of CSF GAP-43 Levels with CSF Core AD Biomarkers

**Figure 2:**
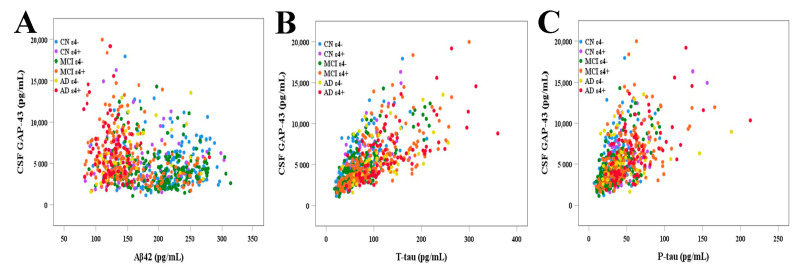
The correlations of CSF GAP-43 with CSF core AD biomarkers, including Aβ42 (**A**), T-tau (**B**) and P-tau (**C**). CSF: cerebrospinal fluid; GAP-43: growth-associated protein 43; Aβ: amyloid-β; T-tau: total tau; P-tau: phosphorylated tau; CN: cognitively normal; MCI: mild cognitive impairment; AD: Alzheimer’s disease.

CSF GAP-43 was correlated with CSF Aβ42 in MCI ε4+ and AD ε4− groups (r_s_ = −0.306, *p* < 0.001; r_s_ = 0.379, *p* = 0.016; respectively), but there were no similar significant relationships found in CN ε4−, CN ε4+, MCI ε4−, and AD ε4+ groups (r_s_ = 0.078, *p* = 0.312; r_s_ = 0.056, *p* = 0.649; r_s_ = −0.094, *p* = 0.180; r_s_ = −0.078, *p* = 0.498; respectively) (Table 2 and Figure 2A). We found there were strong correlations between CSF GAP-43 and T-tau in CN ε4−, CN ε4+, MCI ε4−, MCI ε4+, AD ε4−, and AD ε4+ groups (r_s_ = 0.711, *p* < 0.001; r_s_ = 0.709, *p* < 0.001; r_s_ = 0.751, *p* < 0.001; r_s_ = 0.742, *p* < 0.001; r_s_ = 0.453, *p* = 0.003; r_s_ = 0.696, *p* < 0.001; respectively) (Table 2 and Figure 2B). Similarly, CSF GAP-43 had significant positive correlations with P-tau in CN ε4−, CN ε4+, MCI ε4−, MCI ε4+, and AD ε4+ groups (r_s_ = 0.579, *p* < 0.001; r_s_ = 0.576, *p* < 0.001; r_s_ = 0.581, *p* < 0.001; r_s_ = 0.611, *p* < 0.001; r_s_ = 0.509, *p* < 0.001; respectively), except for AD ε4− group (r_s_ = 0.227, *p* = 0.158) (Table 2 and Figure 2C).

### 3.4. Diagnostic Ability of CSF GAP-43 and CSF Core AD Biomarkers

**Figure 3:**
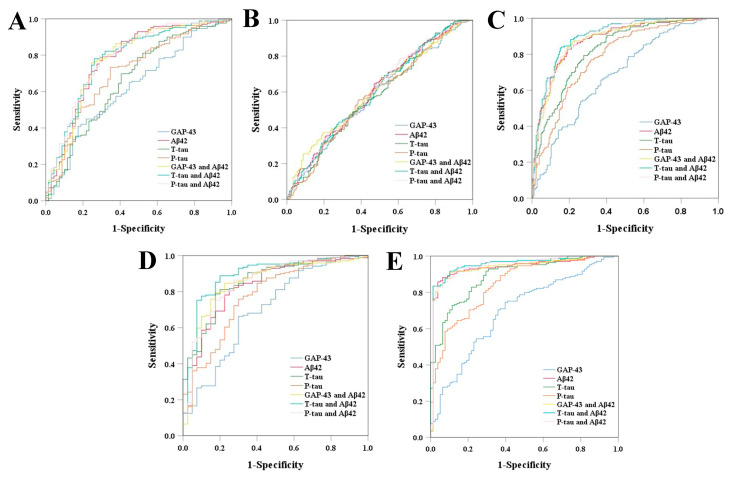
ROCs for the diagnostic accuracy of CSF biomarkers. CN ε4– versus CN ε4+ (**A**), CN ε4– versus MCI ε4– (**B**), CN ε4– versus MCI ε4+ (**C**), CN ε4– versus AD ε4– (**D**) and CN ε4– versus AD ε4+ (**E**). GAP-43: growth-associated protein 43; Aβ: amyloid-β; T-tau: total tau; P-tau: phosphorylated tau.

The AUCs were calculated by performing ROC analyses to test the diagnostic accuracy of CSF GAP-43 and CSF core biomarkers. Compared to CN ε4−, the diagnostic accuracy of these CSF biomarkers was particularly good for MCI ε4+, AD ε4−, and AD ε4+, since the AUCs were obviously larger than that of other diagnoses (Table 3 and Figure 3). The diagnostic performance of CSF GAP-43 was poorer than other CSF biomarkers. After combination with CSF Aβ42, the diagnostic accuracy of CSF GAP-43 had been significantly increased, even showing no significant difference compared with the combination of P-tau and Aβ42 for MCI ε4+ (GAP-43 and Aβ42 versus P-tau and Aβ42, *p*  =  0.071), AD ε4− (GAP-43 and Aβ42 versus P-tau and Aβ42, *p*  =  0.905), and AD ε4+ (GAP-43 and Aβ42 versus P-tau and Aβ42, *p*  =  0.127; Supplementary Table S1).

### 3.5. Cross-Sectional Correlations of CSF GAP-43 with MMSE, Hippocampus Volumes, and Ventricular Volumes

We further explore the relationships between CSF GAP-43 and MMSE, hippocampus volumes, and ventricular volumes at baseline by performing multiple linear regression analysis (Table 4). Significant negative correlations were observed between GAP-43 and MMSE scores in both MCI ε4− and MCI ε4+ groups (β = −0.148, *p* = 0.029; β = −0.179, *p* = 0.014; respectively), which was not found in the other four groups. A higher GAP-43 level was related to smaller hippocampus volumes in CN ε4− and AD ε4− groups (β = −0.117, *p* = 0.036; β = −0.276, *p* = 0.045; respectively). Furthermore, GAP-43 was found to be correlated with smaller ventricular volumes in CN ε4−, MCI ε4−, MCI ε4+, and AD ε4+ groups (β = −0.287, *p* < 0.001; β = −0.139, *p* = 0.027; β = −0.279, *p* < 0.001; β = −0.228, *p* = 0.027; respectively).

### 3.6. Longitudinal Associations between Baseline CSF GAP-43 and MMSE Progression

Next, GEE was utilized to test the associations between baseline CSF GAP-43 level and cognition decline, after adjustment for age, sex, and education. The baseline CSF GAP-43 level was found to be significantly and negatively associated with MMSE scores in both MCI ε4− and MCI ε4+ groups (β  =  −0.197, *p*  = 0.027; β  =  −0.151, *p*  = 0.032; respectively) (Table 4). Longitudinal data analysis was not performed in CN ε4−, CN ε4+, AD ε4−, and AD ε4+ groups, for the reason of a lack of large amounts of follow-up data of these groups.

### 3.7. Ability of CSF GAP-43 to Predict Future Cognitive Impairment

**Figure 4:**
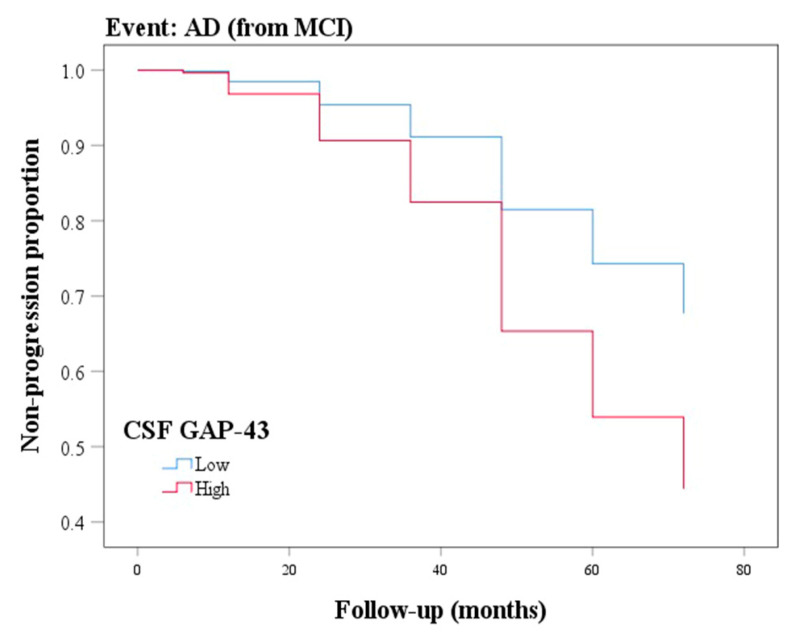
Baseline CSF GAP-43 as a predictor of conversion from MCI to AD. GAP-43 was analyzed as categorical variables using median value in the Cox proportional hazard model, after adjustment for age, sex, and education. GAP-43: growth-associated protein 43; MCI: mild cognitive impairment; AD: Alzheimer’s disease.

We further explored the ability of CSF GAP-43 to predict conversion from MCI to AD by performing the Cox proportional hazard model, after controlling for age, sex, and education. The GAP-43 was treated as a dichotomized variable and the median value of the GAP-43 level was used as a cut-off to calculate the HR. We found the group with high GAP-43 (concentration ≥ 4350.7 pg/mL) progressed to dementia more rapidly compared with the group with lower values (concentration < 4350.7 pg/mL) (HR: 2.079, 95% CI: 1.340−3.226, *p*  =  0.001; Figure 4).

## 4. Discussion

In this study, the main findings were as follows: (1) The CSF GAP-43 level was significantly elevated in MCI ε4+, AD ε4−, and AD ε4+ groups compared with CN ε4− or MCI ε4− group; (2) The CSF GAP-43 level was significantly related to T-tau and P-tau in almost all of the six groups, but the significant relationship with Aβ42 was not found in CN ε4−, CN ε4+, MCI ε4−, and AD ε4+ groups; (3) The diagnostic accuracy of CSF GAP-43 was greatly improved after combined with CSF Aβ42; (4) The CSF GAP-43 level was negatively correlated with MMSE scores at baseline and follow-up in MCI ε4− and MCI ε4+ groups; (5) Baseline GAP-43 was able to predict the clinical progression of MCI to AD.

Previous studies have shown that synaptic loss occurred at the early stage of AD [4], and it had attracted researchers’ attention to the CSF biomarkers of synaptic function, such as the synaptosomal-associated protein 25 (SNAP-25) [22], neurogranin [23], and GAP-43 [19]. To investigate whether the CSF GAP-43 and *APOE* ε4 status correlated with the diagnoses, each group was further dichotomized on the basis of *APOE* ε4 status in this study. We found that the level of GAP-43 was significantly elevated in MCI ε4+ group compared with MCI ε4− group. Furthermore, the GAP-43 level was higher in MCI ε4/ε4 group than MCI ε3/ε4 group, as well as between the AD ε4/ε4 group and the AD ε3/ε4 group, although no statistical differences were observed. Hence, we supposed that CSF GAP-43 could be an early biomarker for cognitive decline and the effects of CSF GAP-43 on cognition may be correlated with *APOE* ε4 status and the number of ε4 alleles. Compared with CN ε4- and MCI ε4- groups, we also found that the AD ε4− group had significantly higher GAP-43 levels (*p* = 0.027, *p* = 0.001, respectively). This phenomenon may be due to the small sample size of the ADε4- group. The number of patients in the AD ε4− group was relatively smaller than in CN and MCI groups, possibly causing bias to some extent. Furthermore, the level of GAP-43 was higher in the AD ε4+ group than in the AD ε4− group, even though no significant difference was found which can be attributed to the small sample size of AD patients.

Our study showed strong positive correlations of CSF GAP-43 with CSF T-tau and P-tau levels in five groups apart from the AD ε4− group, which may due to the small sample size of this group. The results were consistent with prior studies which demonstrated that tau phosphorylation played an important role in leading to synaptic dysfunction [24,25]. Interestingly, the negative correlation between CSF GAP-43 and Aβ42 was only found in MCI ε4+ group. It has been reported that the *APOE* ε4 allele can increase the localization of toxic oligomeric Aβ to synapses [26]. We speculated that the *APOE* ε4 allele played an important role in affecting CSF GAP-43 in the pathogenesis of cognitive decline.

We next found CSF GAP-43 offered moderate diagnostic performance for MCI ε4+, AD ε4−, and AD ε4+. The diagnostic accuracy of CSF GAP-43 was significantly increased after combination with CSF Aβ42 and appeared almost the same as those of the combination of T-tau or P-tau with CSF Aβ42. Previous literature showed that the initial target for Aβ was postsynaptic glutamate receptor trafficking, suggesting Aβ preferred to affect the postsynaptic terminals in the early stage of AD [25,27,28]. As we know, CSF-43 is a presynaptic protein. Therefore, the diagnostic performance of CSF GAP-43 was greatly improved when combined with CSF Aβ42 on account of targeting presynaptic and postsynaptic function, respectively. We supported that CSF GAP-43 may be suitable to be an early diagnostic marker for the presymptomatic stage of AD.

Many studies have demonstrated that the loss of synapses is a major factor involved in cognitive decline in early AD [25,29,30]. Sandelius et al. reported that CSF GAP-43 levels were weakly associated with both at baseline and the annual change of MMSE scores, but neither significant correlations were found in clinical subgroups [19]. In our study, the CSF GAP-43 level was observed to be negatively correlated with MMSE scores at the baseline in MCI ε4− and MCI ε4+ groups, which was also observed in terms of MMSE progression over time using longitudinal data. The inconsistent results may be due to the relatively small sample size of the study conducted by Sandelius et al. Furthermore, we found CSF GAP-43 had a favorable predictive value for the conversion from MCI to AD and there was as yet no relevant research.

There were several limitations in our study. First, the restricted sample selection of the ADNI database may thus have limited the generalizability of the results. Using this database, a significant difference in the percentage of females was found between CNε4+ and AD ε4− groups and the number of samples per group was divergent. It may introduce some bias in statistical analysis which should be taken into consideration for interpreting the data. Second, there were large amounts of missing longitudinal data, such as the MMSE scores in some groups. Third, we concentrated on baseline CSF GAP-43 level to predict cognitive decline and brain atrophy. We did not analyze the effects of the change of GAP-43 level over time on cognition since our initial purpose was to explore the roles of the baseline GAP-43 level. Future studies targeting other different populations and with more complete follow-up data are required to confirm our conclusions.

## 5. Conclusions

In summary, our findings revealed that the CSF GAP-43 level was significantly higher in MCI ε4+, AD ε4− and AD ε4+ groups compared with CN ε4− or MCI ε4− group. There were negative relationships between CSF GAP-43 and MMSE scores at baseline and follow-up in MCI ε4− and MCI ε4+ groups. Baseline CSF GAP-43 can predict the clinical progression from MCI to AD. Thus, CSF GAP-43 may be a promising candidate to screen and track disease progression for AD. We suspected that the effects of CSF GAP-43 in the pathophysiology of cognitive decline may be relevant to *APOE* ε4 status.

## Figures and Tables

**Table 1:** Demographics of included participants at baseline.

Baseline Characteristics	CN ε4– (n = 169)	CN ε4+ (n = 69)	MCI ε4– (n = 204)	MCI ε4+ (n = 184)	AD ε4– (n = 40)	AD ε4+ (n = 78)	*p* Value
Age (years)	73 ± 9 ^d^	71 ± 9 ^e^	72 ± 11 ^e^	71 ± 11 ^a,e,f^	77 ± 13 ^b,c,d^	74.5 ± 10 ^d^	<0.001
Female, N (%)	86 (50.9%)	44 (63.8%) ^e^	93 (45.6%)	81 (44.0%)	12 (30.0%) ^b^	35 (44.9%)	0.013
Education (years)	16 ± 3	17 ± 2	16 ± 4	16 ± 4	16 ± 4	16 ± 4	0.131
MMSE	29 ± 2 ^c,d,e,f^	29 ± 1 ^c,d,e,f^	29 ± 1 ^a,b,e,f^	28 ± 3 ^a,b,e,f^	23 ± 3 ^a,b,c,d^	24 ± 3 ^a,b,c,d^	<0.001
CSF Aβ42 (pg/mL)	215 ± 63 ^b,d,e,f^	159 ± 74.5 ^a,c,f^	205 ± 82 ^b,d,e,f^	140.5 ± 51.5 ^a,c,f^	148 ± 59.5 ^a,c,f^	124.5 ± 29.3 ^a,b,c,d,e^	<0.001
CSF T-tau (pg/mL)	54.2 ± 31.5 ^d,e,f^	63.8 ± 41.2 ^d,e,f^	56.7 ± 41.5 ^d,e,f^	89.75 ± 75 ^a,b,c,f^	106 ± 71.5 ^a,b,c^	120 ± 75.2 ^a,b,c,d^	<0.001
CSF P-tau (pg/mL)	27.5 ± 19.8 ^b,d,e,f^	37.5 ± 24.3 ^a,c,f^	28.6 ± 21.9 ^b,d,e,f^	42.25 ± 30.1 ^a,c^	41.25 ± 32.6 ^a,c^	54.7 ± 32.6 ^a,b,c^	<0.001

Measurement values were expressed by median ± interquartile range. ^a^ =  significant differences from CN ε4–, ^b^ =  significant differences from CN ε4+, ^c^ =  significant differences from MCI ε4–, ^d^ = significant differences from MCI ε4+, ^e^ = significant differences from AD ε4–, ^f^ = significant differences from AD ε4+. CN: cognitively normal; MCI: mild cognitive impairment; AD: Alzheimer’s disease; MMSE: Mini-Mental State Examination; CSF: cerebrospinal fluid; Aβ: amyloid-β; T-tau: total tau; P-tau: phosphorylated tau.

**Table 2:** Correlations of CSF GAP-43 levels with CSF core AD biomarkers.

	CN ε4−		CN ε4+		MCI ε4−		MCI ε4+		AD ε4−		AD ε4+	
	r_s_	*p*	r_s_	*p*	r_s_	*p*	r_s_	*p*	r_s_	*p*	r_s_	*p*
Aβ42	0.078	0.312	0.056	0.649	−0.094	0.180	−0.306	<0.001	0.379	0.016	−0.078	0.498
T-tau	0.711	<0.001	0.709	<0.001	0.751	<0.001	0.742	<0.001	0.453	0.003	0.696	<0.001
P-tau	0.579	<0.001	0.576	<0.001	0.581	<0.001	0.611	<0.001	0.227	0.158	0.509	<0.001

CSF: cerebrospinal fluid; GAP-43: growth-associated protein 43; AD: Alzheimer’s disease; Aβ: amyloid-β; T-tau: total tau; P-tau; CN: cognitively normal; MCI: mild cognitive impairment.

**Table 3:** AUCs of CSF biomarkers for clinical diagnoses.

	GAP-43	Aβ42	T-tau	P-tau	GAP-43 and Aβ42	T-tau and Aβ42	P-tau and Aβ42
CN ε4+	0.627 (0.548−0.705) (*p* = 0.002)	0.776 (0.706−0.847) (*p* < 0.001)	0.664 (0.585−0.743) (*p* < 0.001)	0.708 (0.634−0.782) (*p* < 0.001)	0.778 (0.708−0.849) (*p* < 0.001)	0.782 (0.714−0.850) (*p* < 0.001)	0.789 (0.722−0.856) (*p* < 0.001)
MCI ε4-	0.586 (0.528−0.644) (*p* = 0.004)	0.603 (0.546−0.660) (*p* = 0.001)	0.582 (0.524−0.639) (*p* = 0.007)	0.582 (0.524−0.639) (*p* = 0.007)	0.608 (0.551−0.666) (*p* < 0.001)	0.602 (0.545−0.659) (*p* = 0.001)	0.603 (0.546−0.660) (*p* = 0.001)
MCI ε4+	0.686 (0.631−0.741) (*p* < 0.001)	0.879 (0.843−0.915) (*p* < 0.001)	0.825 (0.783−0.868) (*p* < 0.001)	0.785 (0.738−0.832) (*p* < 0.001)	0.880 (0.844−0.916) (*p* < 0.001)	0.901 (0.870−0.933) (*p* < 0.001)	0.888 (0.854−0.922) (*p* < 0.001)
AD ε4-	0.700 (0.606−0.795) (*p* < 0.001)	0.828 (0.754−0.902) (*p* < 0.001)	0.855 (0.792−0.918) (*p* < 0.001)	0.780 (0.700−0.861) (*p* < 0.001)	0.850 (0.779−0.920) (*p* < 0.001)	0.890 (0.830−0.951) (*p* < 0.001)	0.851 (0.781−0.921) (*p* < 0.001)
AD ε4+	0.695 (0.625−0.765) (*p* < 0.001)	0.937 (0.903−0.972) (*p* < 0.001)	0.889 (0.848−0.931) (*p* < 0.001)	0.847 (0.796−0.898) (*p* < 0.001)	0.939 (0.905−0.973) (*p* < 0.001)	0.956 (0.929−0.982) (*p* < 0.001)	0.948 (0.917−0.979) (*p* < 0.001)

AUC: area under curve; CSF: cerebrospinal fluid; GAP-43: growth-associated protein 43; Aβ: amyloid-β; T-tau: total tau; P-tau: phosphorylated tau; CN: cognitively normal; MCI: mild cognitive impairment; AD: Alzheimer’s disease.

**Table 4:** Correlations of CSF GAP-43 with cognition, hippocampus volume, and ventricular volume.

	CN ε4-		CN ε4+		MCI ε4-		MCI ε4+		AD ε4-		AD ε4+	
	β (95% CI)	*p*	β (95% CI)	*p*	β (95% CI)	*p*	β (95% CI)	*p*	β (95% CI)	*p*	β (95% CI)	*p*
Cross-sectional (MLR)												
MMSE	0.069 (−0.082, 0.221)	0.368	0.176 (−0.052, 0.403)	0.128	−0.148 (−0.280, −0.015)	0.029	−0.179 (−0.321, −0.037)	0.014	−0.281 (−0.617, 0.056)	0.099	0.179 (−0.041, 0.399)	0.109
Bilateral hippocampal volume	−0.117 (−0.227, −0.008)	0.036	0.033 (−0.199, 0.265)	0.775	−0.036 (−0.150, 0.078)	0.535	−0.026 (−0.146, 0.093)	0.663	−0.276 (−0.546, −0.006)	0.045	−0.027 (−0.207, 0.152)	0.762
Ventricular volume	−0.287 (−0.426, −0.147)	<0.001	−0.163 (−0.393, 0.067)	0.161	−0.139 (−0.263, −0.016)	0.027	−0.279 (−0.401, −0.158)	<0.001	−0.283 (−0.597, 0.032)	0.076	−0.228 (−0.430, −0.027)	0.027
Longitudinal (GEE)												
MMSE progression	-	-	-	-	−0.197 (−0.372, −0.022)	0.027	−0.151 (−0.289, −0.013)	0.032	-	-	-	-

CN: cognitively normal; MCI: mild cognitive impairment; AD: Alzheimer’s disease; MLR: multiple linear regression; MMSE: Mini-Mental State Examination; GEE: generalized estimating equation.

## Data Availability

Data used in the preparation of this article were obtained from the Alzheimer’s Disease Neuroimaging Initiative (ADNI) database (adni.loni.usc.edu).

## Supplementary Materials

The following supporting information can be downloaded at: https://www.mdpi.com/article/10.3390/cells12010013/s1, Table S1: The comparisons of CSF biomarkers diagnostic accuracy.

## References

[1] Scheltens P., Blennow K., Breteler M.M., de Strooper B., Frisoni G.B., Salloway S., van der Flier W.M. (2016). Alzheimer’s disease. Lancet.

[2] van der Kant R., Goldstein L.S.B., Ossenkoppele R. (2020). Amyloid-β-independent regulators of tau pathology in Alzheimer disease. Nat. Rev. Neurosci..

[3] Jack C.R., Bennett D.A., Blennow K., Carrillo M.C., Dunn B., Haeberlein S.B., Holtzman D.M., Jagust W., Jessen F., Karlawish J. (2018). NIA-AA Research Framework: Toward a biological definition of Alzheimer’s disease. Alzheimers Dement..

[4] Counts S.E., Alldred M.J., Che S., Ginsberg S.D., Mufson E.J. (2014). Synaptic gene dysregulation within hippocampal CA1 pyramidal neurons in mild cognitive impairment. Neuropharmacology.

[5] DeKosky S.T., Scheff S.W. (1990). Synapse loss in frontal cortex biopsies in Alzheimer’s disease: Correlation with cognitive severity. Ann. Neurol..

[6] Denny J.B. (2006). Molecular mechanisms, biological actions, and neuropharmacology of the growth-associated protein GAP-43. Curr. Neuropharmacol..

[7] Neve R.L., Finch E.A., Bird E.D., Benowitz L.I. (1988). Growth-associated protein GAP-43 is expressed selectively in associative regions of the adult human brain. Proc. Natl. Acad. Sci. USA.

[8] de la Monte S.M., Ng S.C., Hsu D.W. (1995). Aberrant GAP-43 gene expression in Alzheimer’s disease. Am. J. Pathol..

[9] Rekart J.L., Quinn B., Mesulam M.M., Routtenberg A. (2004). Subfield-specific increase in brain growth protein in postmortem hippocampus of Alzheimer’s patients. Neuroscience.

[10] Remnestål J., Just D., Mitsios N., Fredolini C., Mulder J., Schwenk J.M., Uhlén M., Kultima K., Ingelsson M., Kilander L. (2016). CSF profiling of the human brain enriched proteome reveals associations of neuromodulin and neurogranin to Alzheimer’s disease. Proteom. Clin. Appl..

[11] Andersson A., Remnestål J., Nellgård B., Vunk H., Kotol D., Edfors F., Uhlén M., Schwenk J.M., Ilag L.L., Zetterberg H. (2019). Development of parallel reaction monitoring assays for cerebrospinal fluid proteins associated with Alzheimer’s disease. Clin. Chim. Acta.

[12] Corder E.H., Saunders A.M., Strittmatter W.J., Schmechel D.E., Gaskell P.C., Small G.W., Roses A.D., Haines J.L., Pericak-Vance M.A. (1993). Gene dose of apolipoprotein E type 4 allele and the risk of Alzheimer’s disease in late onset families. Science.

[13] Farrer L.A., Cupples L.A., Haines J.L., Hyman B., Kukull W.A., Mayeux R., Myers R.H., Pericak-Vance M.A., Risch N., van Duijn C.M. (1997). Effects of age, sex, and ethnicity on the association between apolipoprotein E genotype and Alzheimer disease. A meta-analysis. APOE and Alzheimer Disease Meta Analysis Consortium. JAMA.

[14] Serrano-Pozo A., Das S., Hyman B.T. (2021). *APOE* and Alzheimer’s disease: Advances in genetics, pathophysiology, and therapeutic approaches. Lancet Neurol..

[15] Dumanis S.B., DiBattista A.M., Miessau M., Moussa C.E., Rebeck G.W. (2013). APOE genotype affects the pre-synaptic compartment of glutamatergic nerve terminals. J. Neurochem..

[16] Aisen P.S., Petersen R.C., Donohue M.C., Gamst A., Raman R., Thomas R.G., Walter S., Trojanowski J.Q., Shaw L.M., Beckett L.A. (2010). Clinical Core of the Alzheimer’s Disease Neuroimaging Initiative: Progress and plans. Alzheimers Dement..

[17] Tierney M.C., Fisher R.H., Lewis A.J., Zorzitto M.L., Snow W.G., Reid D.W., Nieuwstraten P. (1988). The NINCDS-ADRDA Work Group criteria for the clinical diagnosis of probable Alzheimer’s disease: A clinicopathologic study of 57 cases. Neurology.

[18] Shaw L.M., Vanderstichele H., Knapik-Czajka M., Clark C.M., Aisen P.S., Petersen R.C., Blennow K., Soares H., Simon A., Lewczuk P. (2009). Cerebrospinal fluid biomarker signature in Alzheimer’s disease neuroimaging initiative subjects. Ann. Neurol..

[19] Sandelius Å., Portelius E., Källén Å., Zetterberg H., Rot U., Olsson B., Toledo J.B., Shaw L.M., Lee V.M.Y., Irwin D.J. (2019). Elevated CSF GAP-43 is Alzheimer’s disease specific and associated with tau and amyloid pathology. Alzheimers Dement..

[20] Jack C.R., Bernstein M.A., Fox N.C., Thompson P., Alexander G., Harvey D., Borowski B., Britson P.J., Whitwell J.L., Ward C. (2008). The Alzheimer’s Disease Neuroimaging Initiative (ADNI): MRI methods. J. Magn. Reson. Imaging.

[21] Zeger S.L., Liang K.Y. (1986). Longitudinal data analysis for discrete and continuous outcomes. Biometrics.

[22] Brinkmalm A., Brinkmalm G., Honer W.G., Frölich L., Hausner L., Minthon L., Hansson O., Wallin A., Zetterberg H., Blennow K. (2014). SNAP-25 is a promising novel cerebrospinal fluid biomarker for synapse degeneration in Alzheimer’s disease. Mol. Neurodegener..

[23] Kvartsberg H., Duits F.H., Ingelsson M., Andreasen N., Öhrfelt A., Andersson K., Brinkmalm G., Lannfelt L., Minthon L., Hansson O. (2015). Cerebrospinal fluid levels of the synaptic protein neurogranin correlates with cognitive decline in prodromal Alzheimer’s disease. Alzheimers Dement..

[24] Hoover B.R., Reed M.N., Su J., Penrod R.D., Kotilinek L.A., Grant M.K., Pitstick R., Carlson G.A., Lanier L.M., Yuan L.-L. (2010). Tau mislocalization to dendritic spines mediates synaptic dysfunction independently of neurodegeneration. Neuron.

[25] Pereira J.B., Janelidze S., Ossenkoppele R., Kvartsberg H., Brinkmalm A., Mattsson-Carlgren N., Stomrud E., Smith R., Zetterberg H., Blennow K. (2021). Untangling the association of amyloid-β and tau with synaptic and axonal loss in Alzheimer’s disease. Brain.

[26] Spires-Jones T.L., Hyman B.T. (2014). The intersection of amyloid beta and tau at synapses in Alzheimer’s disease. Neuron.

[27] Roselli F., Tirard M., Lu J., Hutzler P., Lamberti P., Livrea P., Morabito M., Almeida O.F. (2005). Soluble beta-amyloid1-40 induces NMDA-dependent degradation of postsynaptic density-95 at glutamatergic synapses. J. Neurosci..

[28] Shemer I., Holmgren C., Min R., Fülöp L., Zilberter M., Sousa K.M., Farkas T., Härtig W., Penke B., Burnashev N. (2006). Non-fibrillar beta-amyloid abates spike-timing-dependent synaptic potentiation at excitatory synapses in layer 2/3 of the neocortex by targeting postsynaptic AMPA receptors. Eur. J. Neurosci..

[29] Terry R.D., Masliah E., Salmon D.P., Butters N., DeTeresa R., Hill R., Hansen L.A., Katzman R. (1991). Physical basis of cognitive alterations in Alzheimer’s disease: Synapse loss is the major correlate of cognitive impairment. Ann. Neurol..

[30] Scheff S.W., Price D.A., Schmitt F.A., DeKosky S.T., Mufson E.J. (2007). Synaptic alterations in CA1 in mild Alzheimer disease and mild cognitive impairment. Neurology.

