# Supplementary material for: Association of CSF GAP-43 and APOE ε4 with Cognition in Mild Cognitive Impairment and Alzheimer’s Disease
Source: Cells. 2022 Dec 21;12(1):13. doi: 10.3390/cells12010013 (PMC9818551; doi:10.3390/cells12010013)
Supplement: Supplementary file 1 [file cells-12-00013-s001.zip › cells-1949640-supplementary.pdf]

Supplementary Material

Table S1. The comparisons of CSF biomarkers diagnostic accuracy.

	MCI ε4+	AD ε4-	AD ε4+
Variables		GAP-43	
Aβ42	$P < 0.001$	0.023	$P < 0.001$
T-tau	$P < 0.001$	$P < 0.001$	$P < 0.001$
P-tau	$P < 0.001$	0.047	$P < 0.001$
Variables		GAP-43 and Aβ42	
T-tau and Aβ42	0.007	0.012	0.028
P-tau and Aβ42	0.071	0.905	0.127

CSF: cerebrospinal fluid; CN: cognitively normal; MCI: mild cognitive impairment; AD: Alzheimer's disease; GAP-43: growth-associated protein 43; Aβ: amyloid-β; T-tau: total tau; P-tau: phosphorylated tau.
